# Ethical Considerations for Wastewater Surveillance Conducted by the US Department of Defense

**DOI:** 10.2196/67145

**Published:** 2025-02-06

**Authors:** Hunter Jackson Smith, Richard T Agans, William J Kowallis

**Affiliations:** 1 Global Emerging Infections Surveillance Branch, Armed Forces Health Surveillance Division, 11800 Tech Rd Suite #200, Silver Spring, MD, 20904, United States, 1 3013193272; 2 Johns Hopkins Berman Institute of Bioethics, Baltimore, MD, United States; 3 Applied Technology and Genomics Division, Defense Centers for Public Health, Dayton, OH, United States; 4 Department of Emerging Biological Threats, Defense Centers for Public Health, Aberdeen, MD, United States; 5 Department of Computational and Chemical Sciences, Carlow University, Pittsburgh, PA, United States

**Keywords:** wastewater, surveillance, ethics, military, Department of Defense

## Abstract

The US Department of Defense (DoD) is establishing its wastewater surveillance capacities to support national security objectives and promote the public health and medical readiness of US service members. Wastewater surveillance is an emerging technology that has traditionally been leveraged for detecting infectious diseases. However, its potential future applications could yield a vast and unpredictable amount of information that could be used for a wide variety of both health- and nonhealth-related purposes. The US military also serves an inimitable role for the country and its citizens, and exercises significant levels of control over its service members compared to civilian organizations. Further, its present and potential wastewater surveillance activities may reach far beyond just military installations. These factors raise unique ethical considerations that must be accounted for by leaders and policymakers to ensure the DoD implements a wastewater surveillance network in a manner that is both impactful in supporting public health and appropriate to the scope and population under surveillance. This paper explores important ethical features in conducting wastewater surveillance that are both specific to the DoD experience and applicable to wider public health initiatives.

## Introduction

Wastewater represents a promising source for obtaining valuable public health data to inform decision-making and response efforts [1]. Wastewater testing includes the tools and technologies used for various activities (eg, research, law enforcement, public health), while wastewater surveillance (WWS) is the activity of assessing biological, chemical, and analyte signatures for determining population-level trends to inform public health decision-making [2-5]. Traditionally, WWS has been leveraged to monitor for select infectious disease pathogens, such as poliovirus and more recently, SARS-CoV-2 during the COVID-19 pandemic [2-4,6]. However, wastewater testing has been used in recent years to detect numerous other targets, ranging from dietary patterns to the genetic makeup of communities [2,3,6-10]. Wastewater surveillance is an appealing public health instrument due to its scalability, cost-effectiveness compared to clinical monitoring, and the ability to detect surges in infectious disease cases, days before they become clinically relevant [11,12]. Recognizing the potential utility and value of WWS, the Centers for Disease Control and Prevention led the development of the National Wastewater Surveillance System. This system allows environmental scientists and public health departments across the country to track specific pathogens, including SARS-CoV-2, influenza A, respiratory syncytial virus, and mpox and report data into the National Wastewater Surveillance System to inform public health decision-making [13]. This system currently collects data from over 1000 sites across the United States, representing 35% of the US population, and it continues to add collaborating sites and other pathogens of public health concern to its surveillance list [13]. There are also plans by researchers and private corporations to further expand the detection capabilities of wastewater testing technologies to include illicit drug use, noncommunicable diseases, genetic information, and other targets. There are meaningful differences in the ethical considerations between wastewater testing and WWS, partly because wastewater testing has broader applications, such as research, law enforcement, and espionage [14,15]. However, this paper focuses on WWS, addressing wastewater testing only as it pertains to WWS as a public health activity.

Recently, the Department of Defense (DoD) has started prioritizing wastewater testing and surveillance, as highlighted in the emerging capabilities and technologies section of the 2023 Biodefense Posture Review [16]. It currently oversees multiple individual WWS projects within the United States and overseas, partners with other government agencies and private industries to explore and expand its wastewater testing technologies, and is building the capacity to develop and manage a DoD WWS network. It has already invested tens of millions of dollars toward advancing its WWS efforts. The DoD plays a distinctive role in ensuring social security and maintains a unique relationship with its ervice members (SM). A DoD WWS network has a potentially vast global scope, covering diverse civilian and military populations [17]. Given (1) the DoD’s unique population and security considerations, (2) the rapid evolution and promising applications of wastewater testing technologies, and (3) the significant unknowns regarding the potential future uses of these technologies, there is an urgent need to address the ethical considerations inherent to implementing a WWS network for the DoD. These efforts are imperative to ensure it is executed appropriately and has suitable policies in place for management.

It is critical that environmental scientists, policymakers, and those engaged in WWS must be aware of the current and potential ethical issues related to wastewater testing and surveillance. Policy development, particularly for emerging technologies, must be focused on ethical implications and should aim to provide appropriate guidance and boundaries. This paper explores the ethical features of WWS and offers recommendations to address these concerns— that are important for any group planning to implement a WWS system. While many ethical considerations discussed in this paper are universal to any WWS system, their application within the DoD (whether in identity, scope, or importance) compared to other contexts is unique and deserves particular attention.

## Targets of Wastewater Surveillance

The scientific evidence and methodology for wastewater sampling and testing must be established for each pathogen or target under consideration for surveillance. Pursuing large-scale sampling without a sufficient understanding of the target would be both ineffective and an unethical use of resources. For infectious diseases, this might involve pathogen-specific knowledge of (1) sampling and detection methods; (2) laboratory testing, genomic sequencing, and analysis; (3) relationship between detection levels in wastewater and clinical detection in humans; and (4) methods to mitigate the spread of the disease. A key component of conducting ethical public health activities includes ensuring the surveillance and responses to the surveillance findings are done in effective, scientifically sound, and evidence-based ways. The negative opportunity costs of inefficiently utilizing public health funds could harm other health areas where funds could have otherwise been allocated. Addressing this concern should include pilot testing of new pathogen targets or wastewater testing technologies as well as cost-effectiveness analyses prior to full integration into a WWS system [18]. For example, if a pilot project discovered that a new potential feature of the WWS system only marginally reduced infectious disease transmission at a significant cost, then those funds might be better directed to more effective public health activities.

Kass [19] describes an ethical framework for the assessment of public health programs and emphasizes that one of the key aspects of an ethically conducted program includes its effectiveness in achieving the stated goals. Relatedly, monitoring and program evaluation should be incorporated into the workflow of a public health program to ensure alignment with its goals [20]. For WWS, this could involve monitoring to determine whether WWS programs are effective at limiting infectious disease transmission among communities where they are being implemented, compared to those without such programs. Additionally, as WWS technologies and methodologies are developed and refined, efforts should be made to assess for concordance between WWS findings and clinical or on-ground surveillance data. Thus, an ethical WWS program must employ scientifically sound sampling and analysis methods and demonstrate effectiveness in achieving its goals, supported by clinical and epidemiologic evidence.

Currently, most WWS programs are focused on infectious diseases. However, there are other potential targets that have been explored beyond infectious pathogens (eg, noncommunicable diseases such as cardiovascular disease and cancer). While it is difficult to predict what technologies will become available in the future to assess WWS [21,22], many of the core ethical issues at present will remain relevant regardless of the future technological capability. The DoD leadership should be cognizant of preventing excessive mission creep (ie, broadening of the original objective) [23,24]. Using WWS for purposes beyond promoting and preserving health can be dangerous and cause ethically fraught scenarios. For example, while opioids and other substances of abuse (eg, methamphetamines, cocaine, nicotine) could be monitored via WWS, it raises equity concerns and risks of overpolicing [25]. Although the DoD already implements randomized drug screening for its SMs, WWS could introduce additional equity concerns. For instance, many higher-ranking personnel and officers typically live off-base, but the DoD would more accessibly conduct WWS in on-base areas where lower-ranking personnel primarily reside. This disparity might result in military leadership focusing punitive actions on lower-ranking enlisted personnel in an inequitable, unjustified manner, despite the possibility that higher-ranking individuals living off-base might use illicit substances in equal measures but remain undetected. This inequity would be particularly problematic considering that WWS captures all individuals using the sanitation system, including SMs, families, civilian employees, contractors, and visitors. In the future, WWS could also be used to detect medication-related metabolites (eg, reproductive health medications), human genetic content (eg, racial or ethnic composition of communities), and other potentially sensitive or politically contentious targets. It is important to thoughtfully consider what may become possible for WWS capabilities and to ensure that DoD leaders and policymakers remain vigilant in keeping its focus on appropriate health targets and public health–promoting outcomes.

## Populations and Areas of Wastewater Surveillance

Where should the DoD focus its wastewater surveillance efforts? This question is practically, legally, and ethically important because there are few areas where WWS could be conducted with only DoD SMs present. Understanding the size and vulnerability of the populations targeted by WWS can influence the interpretation of and responses to the data produced by it [26]. Thus, exploring where the DoD should conduct WWS naturally leads to the question: on whom should the DoD focus its WWS efforts?

On-base DoD locations and DoD transport vessels are obvious areas to focus WWS efforts. Four primary location types can be considered for on-base WWS: (1) facilities, (2) clinics and hospitals, (3) recruit and trainee sites, and (4) housing. On-base DoD locations refer to areas both within the United States and abroad, including permanent international installations and temporary bases established during conflicts. On-base facilities (both medical and nonmedical) and recruit and trainee sites are the least ethically problematic locations, as these areas typically constitute force health protection–relevant activities and have the highest concentration of SMs. While DoD civilian and contractor personnel also work in these areas, conducting WWS at these locations is fully within the DoD’s responsibility and legal jurisdiction to support and promote the health of DoD SMs as well as civilian and contractor personnel. Military transport vessels (eg, large aircraft and naval ships) may prove to be particularly important catchment points for sentinel surveillance, especially for tracking the transmission of infectious diseases [27-29].

On-base housing is another force health protection–relevant area, as these are locations where SMs and those closest to them spend most of their off-duty time. An SM’s family often lives on-base with them, and the family’s health can directly impact the health of SMs and their units. Further, infectious diseases present among SM families are also present among SMs; therefore, conducting surveillance of beneficiaries can better inform the epidemiologic picture of infectious disease prevalence and transmission within military units. Thus, there is a high probability of detecting pathogens of DoD significance in these areas. Moreover, the DoD has a precedent of assuming some fiduciary responsibility for promoting the health and well-being of SM families, through a variety of clinical and social support systems (eg, Tricare health care coverage, paternity/maternity leave policy, education options, free counseling services, housing support, wellness centers). It is also impossible to extricate on-base housing WWS sampling of SMs from family members who live with them. Therefore, it is within the DoD’s purview to promote SM families’ health, and if the WWS is conducted in a minimally invasive, deidentified, and ethically sound manner, WWS could be a valuable way to further support these health objectives.

The DoD also has a vested interest in WWS abroad; these international locations can be grouped into three categories: (1) joint military exercises, (2) ally military installations, and (3) foreign areas of mutual interest with the host nation. There should be two key underlying requirements for all international locations (beyond DoD installations located abroad): (1) the DoD must obtain the permission of the host nation in which they are guests to conduct WWS and (2) the WWS should provide some benefit to that host nation. Ensuring these criteria are fulfilled will promote an equitable relationship between nations and help avoid exploitative or colonialist interpretations of mutually beneficial public health activities. Another critical consideration for the DoD in conducting international WWS is that the host nation should be empowered to exercise as much sovereign control over the public health activities occurring in its own territory as it sees fit. The DoD and the host nation may both have interests in conducting WWS in certain locations in that country; however, the host nation should decide if and how WWS should proceed. The DoD’s role should be supportive, which can manifest in a variety of ways depending on the context and needs of the host nation, including leading the WWS project, funding host nation researchers, providing methodologic input and subject matter expertise, or developing technical and analytical capabilities. Challenges may arise if an ally nation requests WWS support that the DoD is not positioned to support, either on principle or due to resource constraints. International scientific collaborations require agreement from all involved parties. If the DoD does not want to engage, then they are not required to do so, particularly if the DoD has ethical reservations regarding an expansion of the ally nation’s surveillance activities to include controversial targets or populations. If the DoD is supportive of the request but is resource constrained, then it can offer support in other ways (eg, technical consultation, references to other potential collaborators). If the foreign nation’s request involves surveillance targets that are of importance to them but not of interest to the DoD, then the DoD can consider offering low-cost support (eg, information, consultation) in the spirit of preserving and strengthening the relationship. Generally, approaching host nations from the perspective of support, providing the host nation opportunities to lead decision-making, and emphasizing the mutually beneficial aspects of the collaboration will support an equitable partnership.

Joint military exercises serve as collaborative training opportunities for two or more militaries to build cohesiveness, promote mission readiness, and grow camaraderie among allies. In such scenarios, it is acceptable for either the DoD or other militaries to lead WWS efforts, provided all the participating militaries whose wastewater is sampled endorse their permission for WWS to be conducted during the exercise, and for the data to be shared with the sampled parties that participated, if requested. This practice supports the participating military’s autonomy by providing an opportunity for group informed consent, whereby a nation’s military can provide permission or denial for participation of its SMs. For WWS at ally military health care facilities and installations, it is appropriate for the ally nation’s military to lead, with the DoD providing support these efforts as defined by what is agreed upon between the two militaries. The flow of data should be bidirectional and beneficial for both parties for the endeavor to be equitable. For international civilian settings, such as health care facilities, the ally nation’s relevant health organizations (eg, ministries of health, health research institutes) should serve as key partners, and the DoD should support and coordinate with them in the conduct of the surveillance and sharing of data. The host nation’s organization should make efforts to ensure awareness of WWS efforts within the communities involved, especially in specific locations such as health care facilities, and that the facility’s leadership is aware, and approves of the WWS.

## Management of Wastewater Surveillance Data

Once the WWS has been conducted, how should data be managed? The DoD faces important ethical considerations regarding the type of data to be collected and its management. A balance must be struck between allowing beneficial data access and ensuring data protection. WWS can generate a variety of data, including molecular and biological signatures, health and disease markers, biochemical indicators of illicit activities, and associated identifiable demographic metadata [17]. While possessing large amounts of data is typically beneficial for determining appropriate responses, course of actions, and preventive planning, protecting these data is crucial, especially as the DoD arguably experiences more frequent and powerful attacks on its information security infrastructure compared to other organizations. Therefore, managing the focus on which data to protect (eg, personally identifiable information vs aggregate data) and how to protect it (eg, cloud or servers vs hardline firewalls) will be crucial for ensuring minimized risk of data weaponization or leakage. Wastewater surveillance can produce highly granular data that can be used by public health researchers and officials for improving the quality of the communities they serve; however, this level of detail is not always essential when communicating with end users. Establishment of well-defined data-sharing agreements which incorporate ethical guidelines and treat WWS data with the same scrutiny as Controlled Unclassified Information or Classified determinations, wherein raw information sharing is conducted only as needed, will aid in reducing improper use of WWS information. Data security can be handled in a similar manner by escalating access and storage restrictions corresponding to data aggregation and specificity. For instance, aggregated, community-level detection data of public health targets could be stored on shared drives with minimal restrictions; however, individual molecular and genetic data could be stored behind hardline firewalls on dedicated IT systems. Anonymization and the use of beacons are additional potential methods that could help in ensuring individual human subjects cannot be identified by WWS; however, both methods have their inherent limitations [30]. Nonetheless, it is likely that data security approaches will not lend to one size fits all solutions and will be dictated by the surveillance effort, parties involved, and required outcomes. The DoD must take strong measures to ensure only authorized personnel access appropriate levels of WWS data.

As WWS data becomes more granular and specific, the ethical responsibility to ensure data security and appropriate use and access increases proportionally. This escalation of responsibility in proportion to the level of granularity of the data is derived from the increasing probability of it being personalized and potentially damaging to individuals or small groups. For example, depending on where the wastewater is sampled, it could be possible to pinpoint illicit drug use or the location of an individual to a specific household. In fact, analyzing local sewage for genetic markers was one of the methods potentially used by the United States to confirm Osama bin Laden’s location prior to him being killed in 2011 [15]. It may also become possible in the future to obtain personal genetic information through WWS and to associate it with personally identifiable information; the DoD must be prepared to either dispose or strongly protect this data. There are significant risks if foreign adversaries gain access to this level of data, as well as real concerns regarding how this information could be misused domestically in politically motivated ways. One way to combat these concerns is to establish clear sampling strategies with requirements to destroy unused sample portions once the appropriate predetermined targets have been tested or detected.

There are also important considerations regarding the sharing of WWS data when it has broader implications beyond the DoD, and is relevant to other public health entities, such as US government groups (eg, local health departments, the Centers for Disease Control and Prevention), ally nations, or nongovernmental organizations (eg, the Bill and Melinda Gates Foundation). There are certain ethical responsibilities in the sharing of appropriate levels of data when it can also be of benefit to others, and the DoD should take steps to collaborate with these groups to promote health security. Thus, taking all these considerations into account, one overarching goal for a DoD WWS network is to ensure the data is maintained at an aggregate level as much as possible for monitoring of community and population-level trends rather than specific information on individuals or small groups.

## Utilization and Response to Wastewater Surveillance Data

Public health surveillance data should not be collected unless there is a potential for a meaningful and actionable response. If surveillance findings are not translated to public health action or policy changes, the benefits accrued from the surveillance efforts are minimal [31]. Collecting data only for the sake of collection is an inappropriate use of resources; therefore, there should be focused and organized plans in place for using the WWS systems and information derived from them prior to their implementation. Additionally, there should be rigorous discussions about how military leaders and public health officials respond to such information as it becomes available to them. There are innumerable ways in which the leadership could respond to WWS data when it is received in near real-time. While this information offers an opportunity to ethically use that data, there is a risk of inappropriate response to it [32]. Notably, military commanders make the final decisions regarding public health actions or interventions, particularly those of consequence. It is likely that a medical or public health professional will not be making the decision, and the military commander may have no background in science, medicine, or public health. While making the intervention decisions in response to WWS data, these commanders and their leadership teams should lean heavily upon the expert advice of medical and public health professionals to inform their decisions to help ensure they take the most appropriate actions. The key consideration here is that the responses—whether determined by military command or leadership from preventive medicine, public health, or clinical groups—should be appropriate, proportional, and context- and community-specific based on the information received through the WWS system.

## General Ethical Considerations

Finally, there are unique ethical considerations for the DoD that civilian organizations do not necessarily face to the same extent. The military has substantial authority, control, and access over the lives of its SMs. It can dictate aspects ranging from what to wear, where to live, how to behave, and nearly every aspect of SMs’ lives. Given the extent of this control and its durability and depth, the DoD has an elevated ethical responsibility toward the health and well-being of its SMs [33]. Further, there is a public health ethics principle, which argues that a public health program should use the least invasive or minimally burdensome (to the population being targeted by the program) methods to accomplish its goals [16,34]. Service members already forgo significant autonomy afforded to civilians; therefore, any public health program that might additionally strip away their autonomy must be seriously deliberated before implementation. Although WWS may not directly interfere with the lives of SMs while the data is collected, the actions related to WWS (eg, how leadership responds to the information, what data is collected, and how the data is managed) could infringe upon their privacy and autonomy. However, if conducted appropriately, the potential health benefits for the SMs would likely outweigh minor privacy or autonomy concerns. Additionally, the implications extend beyond military populations when considering the health of SMs. The mission of the DoD and SMs is to protect US citizens, interests, and national security. If SMs are unwell, experience disease outbreaks, or are targeted using bioweapons, it places the country at risk, as they will be less able to support their security objectives by failing to achieve optimal operational capabilities and mission readiness. Furthermore, SMs and military installations do not exist in isolation. If an infectious disease emerges among a military population in a certain area, it is likely there is a similar problem in the surrounding civilian community, or at least presents a risk to them. Therefore, these considerations add to the elevated responsibility of the DoD to support and promote the health of its SMs.

There are also several broad ethical considerations for how the DoD conducts WWS. There is a tension underlying many public health actions, between preserving personal freedom and autonomy of individuals and maximizing the public health benefits for the group. This tension is manifested in several ways, including the amount and types of data to be collected, proportionality and appropriateness of response to the data, level of invasiveness of the surveillance, populations, and areas selected for surveillance [35,36]. DoD leadership should balance protecting the freedom and autonomy of its SMs while also prioritizing the health of the force from a population perspective [33]. Additional resources providing ethical guidance for the operation of public health surveillance systems that are applicable for WWS are available from the American Public Health Association’s Public Health Code of Ethics and the World Health Organization’s Guidelines on Ethical Issues in Public Health Surveillance [34,37].

Justice is another key ethical concept, and when appraising a DoD WWS program, two relevant perspectives must be considered: (1) distributive justice and (2) procedural justice. Distributive justice is an ethical principle concerned with the fair distribution of benefits and burdens. In the DoD WWS context, it involves ensuring that the distribution of resources, outcomes, and interventions based on the surveillance findings is done in an equitable way (eg, distributing vaccines based on WWS findings of acute elevations in vaccine-preventable diseases). It also includes the fair distribution of resources for conducting WWS throughout the United States and international installations. Procedural justice is an ethical principle concerned with fair processes and methods. It includes features such as inclusion, transparency, neutrality, respect, and trust [38]. For DoD WWS, it might involve transparent communications and decision-making, community considerations, stakeholder participation, access to relevant data for the surveilled communities, among others.

There are a variety of other general ethical considerations that are applicable to WWS, which are briefly mentioned here and should be explored further. First, there are differences between conducting public health surveillance versus research and the varying regulatory, oversight, and ethical requirements. Second, there may be differences in what actions are permissible when considering baseline surveillance versus those needed during public health emergencies. Finally, there are inevitable future possibilities beyond our current capabilities or knowledge regarding what might become possible through conducting WWS. What might WWS be capable of in the future, and how do we anticipate and plan for that? Although there are myriad ethical considerations for the appropriate conduct of WWS by the DoD both currently and in the future, it is undoubtedly a burgeoning area of public health that the DoD should pursue and be engaged in a substantial, effective, and meaningful manner, as there are innumerable potential benefits to a DoD WWS network.

## Conclusions

Wastewater surveillance offers tremendous promise and opportunities to advance public health by enhancing disease surveillance, informing public health and clinical decision-making, and improving public health response times to mitigate negative health impacts to populations [39,40]. The DoD should adopt WWS and position itself to take advantage of the current and potential future benefits. It should also recognize the practical and ethical differences between using wastewater testing for public health surveillance, research, or other potential military purposes. With over 1.3 million active-duty SMs, 1.1 million National Guard and Reserve members, and 700,000 civilian personnel, the DoD is the largest organization in the United States, and has responsibilities to their health and readiness [41]. Further, the Military Health System serves a total of approximately 9.5 million beneficiaries, which includes both SMs and their families [42]. WWS has the potential to benefit not only these populations, but others in the United States and overseas as well. However, these opportunities also present a potential for misuse, which could be detrimental to those being surveilled, raising important ethical questions about how and why WWS should be conducted. The DoD also has elevated ethical obligations to the well-being of its SMs due to the breadth and depth of control it exercises over them. Although ethical issues may arise related to WWS, these issues can and should be addressed through proper deliberation and policy implementation, as the potential public health benefits could be substantial. The DoD has responsibilities to protecting the national security of its citizens broadly, which implies a crucial role in infectious disease surveillance for outbreaks as well as bioengineered and emerging pathogen threats. Therefore, the DoD should pursue wastewater surveillance in an ethically appropriate, scientifically robust, and thoughtfully planned and executed manner to guide public health decision-making, protect the well-being of its SMs, and support US government health security objectives.

## Abbreviations

DoD: Department of Defense
SM: Service member
WWS: wastewater surveillance

## References

[1] Diamond MB, Keshaviah A, Bento AI (2022). Wastewater surveillance of pathogens can inform public health responses. Nat Med.

[2] Huizer M, Ter Laak TL, de Voogt P, van Wezel AP (2021). Wastewater-based epidemiology for illicit drugs: a critical review on global data. Water Res.

[3] Kilaru P, Hill D, Anderson K (2023). Wastewater surveillance for infectious disease: a systematic review. Am J Epidemiol.

[4] Luo J, Bello D, Pagsuyoin S (2023). Long-term wastewater-based surveillance and impacts of the COVID-19 pandemic on drug use trends in a U.S. northeast rural town. Science of The Total Environment.

[5] National Academies of Sciences, Engineering, and Medicine, Health and Medicine Division; Division on Earth and Life Studies, Board on Population Health and Public Health Practice, Water Science and Technology Board, Committee on Community Wastewater-based Infectious Disease Surveillance (2023). Wastewater-Based Disease Surveillance for Public Health Action.

[6] Bowes DA, Driver EM, Savic S (2023). Integrated multiomic wastewater-based epidemiology can elucidate population-level dietary behaviour and inform public health nutrition assessments. Nat Food.

[7] Clark M, Severn M (2023). Canadian Journal of Health Technologies.

[8] Choi PM, Tscharke B, Samanipour S (2019). Social, demographic, and economic correlates of food and chemical consumption measured by wastewater-based epidemiology. Proc Natl Acad Sci U S A.

[9] Evans ED, Dai C, Isazadeh S, Park S, Ratti C, Alm EJ (2020). Longitudinal wastewater sampling in buildings reveals temporal dynamics of metabolites. PLoS Comput Biol.

[10] Yang Z, Xu G, Reboud J, Kasprzyk-Hordern B, Cooper JM (2017). Monitoring genetic population biomarkers for wastewater-based epidemiology. Anal Chem.

[11] Farkas K, Hillary LS, Malham SK, McDonald JE, Jones DL (2020). Wastewater and public health: the potential of wastewater surveillance for monitoring COVID-19. Curr Opin Environ Sci Health.

[12] Hart OE, Halden RU (2020). Computational analysis of SARS-CoV-2/COVID-19 surveillance by wastewater-based epidemiology locally and globally: feasibility, economy, opportunities and challenges. Sci Total Environ.

[13] (2023). National wastewater surveillance system (NWSS). Centers for Disease Control and Prevention.

[14] (2024). National wastewater drug monitoring program reports. Australian Criminal Intelligence Commission.

[15] Allison G (2012). How it went down. TIME.

[16] (2023). 2023 Biodefense Posture Review. US Department of Defense.

[17] Goldberg Z, Linder AG, Miller LN, Sorrell EM (2024). Wastewater collection and sequencing as a proactive approach to utilizing threat agnostic biological defense. Health Secur.

[18] Sanjak JS, McAuley EM, Raybern J (2024). Wastewater surveillance pilot at US military installations: cost model analysis. JMIR Public Health Surveill.

[19] Kass NE (2001). An ethics framework for public health. Am J Public Health.

[20] German RR, Lee LM, Horan JM (2001). Updated guidelines for evaluating public health surveillance systems: recommendations from the Guidelines Working Group. MMWR Recomm Rep.

[21] Amin V, Bowes DA, Halden RU (2023). Systematic scoping review evaluating the potential of wastewater-based epidemiology for monitoring cardiovascular disease and cancer. Sci Total Environ.

[22] Ladyzhets B (2024). What toilets can reveal about COVID, cancer and other health threats. Nature New Biol.

[23] Doorn N (2022). Wastewater research and surveillance: an ethical exploration. Environ Sci (Camb).

[24] Rinde M, Science History Institute (2023). The murky ethics of wastewater surveillance. Distillations Magazine.

[25] Hrudey SE, Silva DS, Shelley J (2021). Ethics guidance for environmental scientists engaged in surveillance of wastewater for SARS-CoV-2. Environ Sci Technol.

[26] Coffman MM, Guest JS, Wolfe MK (2021). Preventing scientific and ethical misuse of wastewater surveillance data. Environ Sci Technol.

[27] Ahmed W, Bertsch PM, Angel N (2020). Detection of SARS-CoV-2 RNA in commercial passenger aircraft and cruise ship wastewater: a surveillance tool for assessing the presence of COVID-19 infected travellers. J Travel Med.

[28] (2023). Traveler-based genomic surveillance for early detection of new SARS-cov-2 variants. Centers for Disease Control and Prevention.

[29] Li J, Hosegood I, Powell D (2023). A global aircraft-based wastewater genomic surveillance network for early warning of future pandemics. Lancet Glob Health.

[30] Jacobs D, McDaniel T, Varsani A, Halden RU, Forrest S, Lee H (2021). Wastewater monitoring raises privacy and ethical considerations. IEEE Trans Technol Soc.

[31] Lee LM, Thacker SB (2011). Public health surveillance and knowing about health in the context of growing sources of health data. Am J Prev Med.

[32] Gable L, Ram N, Ram JL (2020). Legal and ethical implications of wastewater monitoring of SARS-CoV-2 for COVID-19 surveillance. J Law Biosci.

[33] Smith HJ (2022). Ethical responsibilities of a military to the social determinants of health of its service members. Mil Med.

[34] (2019). Public health code of ethics. American Public Health Association.

[35] Marckmann G, Schmidt H, Sofaer N, Strech D (2015). Putting public health ethics into practice: a systematic framework. Front Public Health.

[36] Schröder-Bäck P, Duncan P, Sherlaw W, Brall C, Czabanowska K (2014). Teaching seven principles for public health ethics: towards a curriculum for a short course on ethics in public health programmes. BMC Med Ethics.

[37] (2017). WHO guidelines on ethical issues in public health surveillance. World Health Organization.

[38] Bowes DA, Darling A, Driver EM (2023). Structured ethical review for wastewater-based testing in support of public health. Environ Sci Technol.

[39] Keshaviah A, Diamond MB, Wade MJ, Scarpino SV, Global Wastewater Action Group (2023). Wastewater monitoring can anchor global disease surveillance systems. Lancet Glob Health.

[40] Levy JI, Andersen KG, Knight R, Karthikeyan S (2023). Wastewater surveillance for public health. Science.

[41] The executive branch. White House Archives.

[42] (2023). Patients by beneficiary category. Health.mil: The official website of the Military Health System.

